# Alcoholism; Its Treatment from an Analysis of One Hundred and Twenty-five Cases

**Published:** 1879-12

**Authors:** E. C. Helm

**Affiliations:** House Physician; Mercy Hospital, Chicago


### Article VII.

**Alcoholism; its Treatment from an Analysis of One Hundred and Twenty-five Cases (Records of which are to be Found in Mercy Hospital.) By E. C. Helm, m.d., House Physician.**

Therapeutically it has been found convenient to divide alcoholism into six classes:

1. Those cases in which the most prominent symptoms are insomnia, restlessness and anorexia.
2. Ditto with considerable gastric disturbance.
3. Delirium ebrosia.
4. Alcoholic stupor.
5. Cases with the premonitory symptoms of delirium tremens, characterized by anorexia, insomnia, restlessness, tremulousness, illusions and hallucinations.
6. Delirium tremens.

Of the nationalities represented, one-half were Irish, one-fourth were American. The rest were of various nationalities. The number of married to unmarried was about equal. The relation of males as to females was as ten to one. Their ages ranged from twenty-two to seventy-eight. The average age was between thirty-seven and thirty-eight. The first two classes comprised about forty per cent. of the cases. The third and fourth classes about five per cent. each. The fifth class comprised fully thirty-five per cent. of the cases. The sixth class, the remainder, or fifteen per cent.

The first item of treatment in these cases was the withdrawal of alcohol in every form (though a small proportion of the cases, who occupied private rooms, insisted upon having from one to three small portions of brandy or its equivalent, during the first day, and as their demands were, in many cases, seconded by their friends, they were given the brandy, but *only* for the *first* day).

The first class of patients were put in bed, in a quiet room, and given

| | | |
|---|---|---|
| Pot. brom | 1 | 3 |
| Tr. digital | | 25 |

every three hours. If, as their usual bedtime approached, they were still restless and uneasy, they were given chloral hyd. grms. 0.6 to 1.0 every half hour until three doses had been taken. This usually insured sleep. Should it not, they were given no more chloral before midnight, and then but one, or, at most, two doses.

The second class of cases were treated in much the same way, except that in addition to the above treatment they were given 4 grms. of

| | | |
|---|---|---|
| ℞ Acid carbolici | | 4 |
| Tr. gelsemini | | |
| Glycerinæ āā | 15 | |
| Tr. opii. camph | | |
| Aquæ āā | 50 | |

before meals.

If there was very much pain in the epigastrium a sinapism usually gave relief. In not one instance did delirium tremens follow either of these classes of cases. The third class of cases were given bromide, chloral and digatalis, with cold to the head, and were kept quiet, even if their arms and legs had to be pinioned. It was, at times, necessary to give the medicines by the rectum or through an œsophageal tube.

These cases usually quieted down in a few hours, and sank into a heavy sleep; upon awakening, they usually passed into either the first, second or fifth class. Very rarely did it occur that delirium ebrosia was followed by delirium tremens.

The fourth class were let alone, merely kept quiet, and given an abundance of pure air. Upon awakening, they usually passed into either the first, second or fifth class (though a few cases passed into delirium tremens), and were treated accordingly.

The fifth class were treated as if in the first class. Sleep was nearly always induced by the before-mentioned remedies. Instead of sleeping, a few patients perspired profusely with a cold, clammy skin, a weak, frequent, easily-compressible pulse, etc., being that peculiar condition bordering on collapse, in which most of the text-books assert that alcohol is imperatively demanded. Be that as it may, it is a fact that arom. spts. of ammon. (or ammon. carb.) with camphor, *always* produced the desired effect, and that very speedily. After arousing them, the bromides and digitalis would be continued, and with the chloral at night, pushed until sleep was obtained. In not a single case was this treatment followed by delirium tremens or any other unfavorable result. Eighty per cent. of the cases of delirium tremens were in that condition when first seen, and instead of the delirium tremens following the withdrawal of alcohol, it was found that in all these cases they continued to drink until delirium tremens ensued (except in those few cases, i. e., the remaining 20 per cent.), in which the drunken stupor or delirium ebrosia terminated in delirium tremens. In patients with delirium tremens, bromides, chloral and digitalis were freely used, i. e., 1 grm. to 1.3 grm. every two hours until sleep was induced.

There were four deaths in these one hundred and twenty-five cases. One death resulted from an overdose of chloral hyd. which the patient took when the attendant's back was turned. This occurred but a few hours after entering the hospital. The second fatal case was one with acute capillary congestion of the brain and lungs. In this case death occurred a few hours after entering the hospital, indeed, the patient was almost moribund when first seen.

The third case had delirium tremens for several days before entering the hospital. This soon became so violent as to simulate acute mania, if, indeed, it did not terminate in acute insanity. Though he was closely confined in his cell, it was with the utmost difficulty that the nurses could force him to take the medicines. He died the second or third day after entering the hospital.

The fourth and last case of death was a man sixty-five years of age, the last years of whose life had been a continual debauch. He had had delirium tremens many times. He died nearly a month after entering, and with the symptoms of typhoid asthenia.

In all these cases, the bromides were used freely, with no unpleasant results. No bad effects were found to result from digitalis. In 1 grm. doses it exerted a wonderfully quieting effect upon the circulation, the heart promptly yielding to its influence by becoming slower and stronger.

Chloral hydrate was found to act more uniformly as a hypnotic in cases of insomnia from alcohol than its rather uncertain nature would have indicated.

Yet alarming symptoms occasionally supervened when 2 grm. doses were taken. Therefore, of late, doses greater than one gram had seldom been given. Chloral, when given at night, was usually given in one gram doses every half hour until three or four doses had been taken. When thus given it was beneficial and gave rise to no unpleasant effects. The value of chloral has been found to be secondary to that of digitalis or the bromides. It will be seen from these cases that there has been no "tapering off" on the supply of alcohol (as in those comparatively few cases in which it was given, it was given but for *one* day, which could hardly be called "tapering off").

The promptness with which these cases have yielded to this course of treatment, has been extremely gratifying, not only to the physicians, but also to the patients.

The majority of the patients, when entering, think they can hardly survive without "tapering off." Many and bitter are their complaints, but after the first day or two they cease to ask for liquors, and many have said afterward "that they never recovered from a debauch as quickly before."

The average duration of their time in the hospital has been nine days, but by eliminating five cases who, for other reasons remained from one to three months, it will cut this average down to seven days.

MERCY HOSPITAL, CHICAGO, Nov. 20, 1879.

---

AN interesting article on the action of pyrogallic acid in lesions of the skin and mucous membranes, from the pen of Dr. Rosa H. Engert, of this city, appears in the *Wiener Medizinische Wochenschrift*, Oct. 11, 1879, p. 1084.

